# Systems biology network-based discovery of a small molecule activator BL-AD008 targeting AMPK/ZIPK and inducing apoptosis in cervical cancer

**DOI:** 10.18632/oncotarget.3513

**Published:** 2015-03-10

**Authors:** Leilei Fu, Shouyue Zhang, Lan Zhang, Xupeng Tong, Jin Zhang, Yonghui Zhang, Liang Ouyang, Bo Liu, Jian Huang

**Affiliations:** ^1^ State Key Laboratory of Biotherapy, Collaborative Innovation Center of Biotherapy, West China Hospital, Sichuan University, Chengdu, China; ^2^ School of Traditional Chinese Materia Medica, Shenyang Pharmaceutical University, Shenyang, China; ^3^ School of Pharmacy, China Pharmaceutical University, Nanjing, China; ^4^ Collaborative Innovation Center for Biotherapy, Department of Pharmacology & Pharmaceutical Sciences, School of Medicine, Tsinghua University, Beijing, China

**Keywords:** Systems biology network, Apoptosis, AMPK, ZIPK, Dual-target activator (BL-AD008)

## Abstract

The aim of this study was to discover a small molecule activator BL-AD008 targeting AMPK/ZIPK and inducing apoptosis in cervical cancer. In this study, we systematically constructed the global protein-protein interaction (PPI) network and predicted apoptosis-related protein connections by the Naïve Bayesian model. Then, we identified some classical apoptotic PPIs and other previously unrecognized PPIs between apoptotic kinases, such as AMPK and ZIPK. Subsequently, we screened a series of candidate compounds targeting AMPK/ZIPK, synthesized some compounds and eventually discovered a novel dual-target activator (BL-AD008). Moreover, we found BL-AD008 bear remarkable anti-proliferative activities toward cervical cancer cells and could induce apoptosis by death-receptor and mitochondrial pathways. Additionally, we found that BL-AD008-induced apoptosis was affected by the combination of AMPK and ZIPK. Then, we found that BL-AD008 bear its anti-tumor activities and induced apoptosis by targeting AMPK/ZIPK *in vivo*. In conclusion, these results demonstrate the ability of systems biology network to identify some key apoptotic kinase targets AMPK and ZIPK; thus providing a dual-target small molecule activator (BL-AD008) as a potential new apoptosis-modulating drug in future cervical cancer therapy.

## INTRODUCTION

Apoptosis is a complex but highly defined cellular program of demolition with numerous links to many pathological processes, such as cancer [1, 2]. Complexity of apoptosis may inspire systems biology approaches to uncover its inner mechanisms by some mathematical models, such as ordinary differential equations, Petri nets, Bayesian networks, and Boolean model [3, 4]. Hitherto, new emerging therapeutic strategies have been considered to mediate cell death by activating core apoptotic pathways, as well as to remodel the structure of apoptotic network [5]. Therefore, a comprehensive knowledge of protein-protein interaction (PPI) networks may provide a basic framework for better understanding of apoptosis as an integrated system [6, 7]. With increasing genome-wide data of genetic, functional and physical interactions, a robust mathematical model which is well-suited for integrating disparate types of data, seems to be imperative for inferring the apoptotic process [8]. Moreover, inactivation of pro-apoptotic proteins or up-regulation of anti-apoptotic proteins may result in unchecked growth of cells and thus ultimately leading to carcinogenesis [9-11].

Carcinogenesis is a multi-step process caused by genetic alterations involving mutations of some oncogenes or other tumor suppressors that may drive the progressive transformation of normal cells into malignant ones [12]. At systems level, genetic mutations may alter translated proteins and thus disrupting downstream signaling pathways and even the PPI network; ultimately resulting in resistance to apoptosis [13]. Recently, protein kinases has been reported to orchestrate the activation of signaling cascades in response to extracellular and intracellular stimuli to control cell growth, proliferation, survival and apoptosis [14, 15]. Hitherto, cancer drug discovery has significantly benefited from a rapid progress for further understanding how to target the key protein kinases with small molecule compounds in cancer therapy [16]. However, complexity of the apoptotic kinase network may inspire more systems biology approaches to uncover novel kinase targets for cancer drug design [16]. Thus, these findings would provide a key clue for the discovery of novel anti-tumor candidate drugs targeting some key apoptotic kinases.

In this study, we demonstrated the ability of systems biology network to identify some key apoptotic kinase targets, such as AMPK and ZIPK in cervical cancer, and thus provided a small molecule activator (BL-AD008) as a new potential anti-tumor drug in cervical cancer therapy.

## RESULTS

### Construction of core apoptotic kinase network

**Figure 1:**
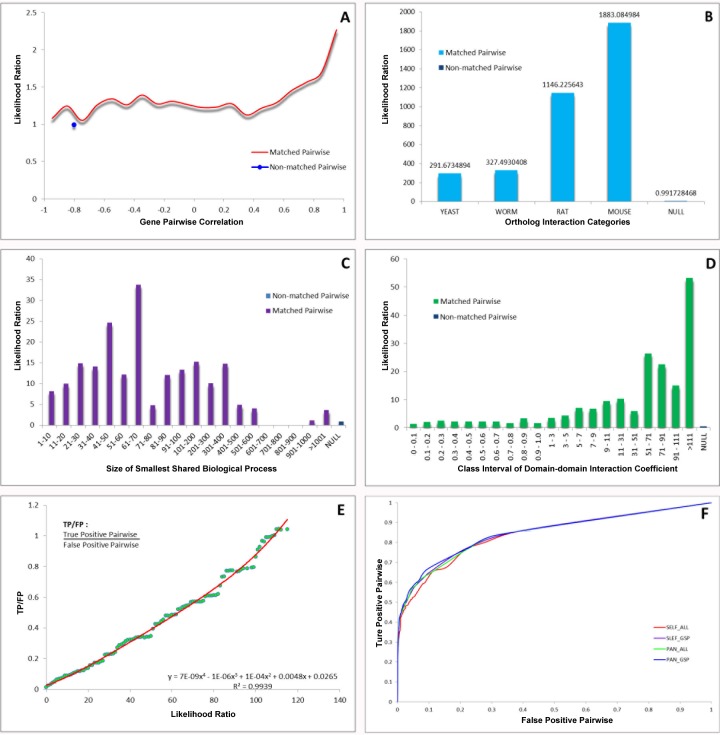
Integration of biological data and network model evaluation (A) Large-scale microarray datasets. (B) The homology analysis. (C) The SSBP for measuring the biological process similarity of a pair of proteins. (D) DDI confidence level from the database Pfam. (E) TP/FP ratios at different Likelihood cutoffs. (F) ROC curves for evaluating the performance of Naïve Bayesian model.

**Figure 2:**
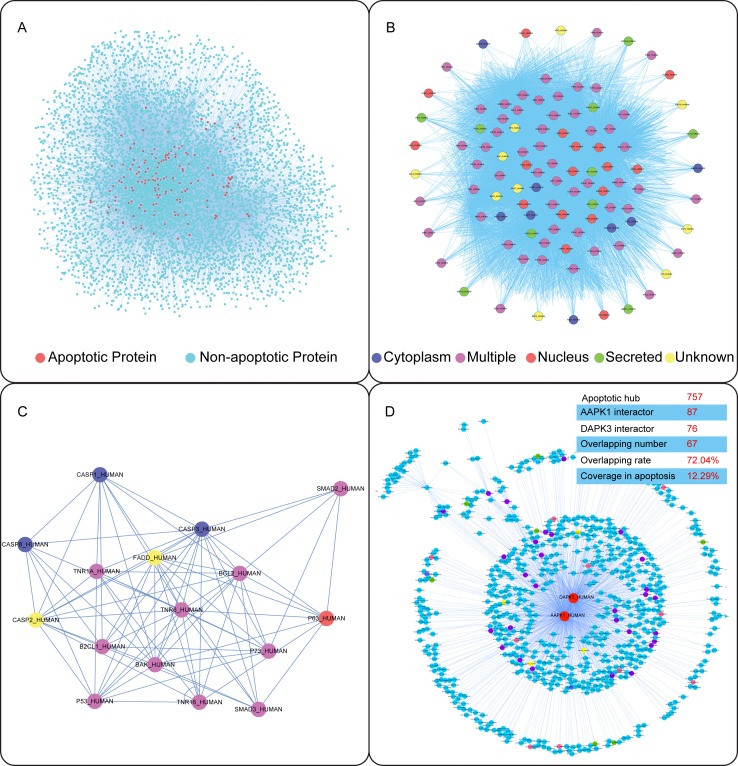
Network-based identification of classical and novel apoptotic (kinase) pathways (A) The global PPI network in apoptosis. (B) Core apoptotic signaling subnetwork. (C) Predicted interactions amongst classical hub proteins in apoptosis. (D) Novel apoptotic kinase pathways involved in AMPK/ZIPK regulation in cancer.

Based upon some online databases, we computationally constructed the global PPI network. To construct the set of true-positive gene pairs, we manually derived physical PPIs. A total number of 85,083 unique PPIs among 13,128 proteins were prepared as data sources for our Golden Standard Positive (GSP) set. We generated a Golden Standard Negative (GSN) set that could be defined as all the possible pair-wise combinations, in which one protein is assigned to the plasma membrane and the other to the nucleus according to GO cellular component annotation, resulting in 23,169,177 pairs in our GSN (Table S1). Moreover, we integrated four different types of biological datasets and chose the likelihood ratio (LR) =117 as the reliability of individual dataset for inferring the apoptotic PPIs. Each dataset could be divided into several bins due to their intrinsic characters, and LR for each bin was calculated, indicating the corresponding results of cross-species interolog mapping (Figure 1A), gene co-expression profiles (Figure 1B), domain-domain interaction (DDI) (Figure 1C) and smallest shared biological process (SSBP) (Figure 1D), respectively. Subsequently, we used LR cutoff as 117 and achieved the global PPI network with 12,809 binary PPIs by combining the prediction set and the positive set (Figure 1E). Using the lunched Naïve Bayesian model, we found that STS containing 12,809 interacting protein pairs conformed by 4,818 unique proteins was input the network model, resulting the area under ROC curve (Figure 1F). As a result, we got the global PPI network, and further modified this network into the apoptotic PPI network (Figure 2A). We identified hub proteins implicated in core apoptotic pathways according to the four golden standards (the degree of each protein, the link number of apoptotic protein, network topology, and significance analysis of microarrays analysis). Thus, combination of the four standards that can be integrated into a well-suited approach to decrease the false-positive PPIs on some level; thereby, confirming apoptotic hub proteins (Figure S1).

### Identification of two key apoptotic kinase targets AMPK and ZIPK

Then, we identified a few of apoptotic hub proteins and their relevant signaling pathways that could be further integrated into the predicted core apoptotic network which composed of 109 proteins (3,767 protein pairs) (Figure 2B) (Table S2). From the core apoptotic network, we identified not only classical hub proteins such as caspases (CASP1/2/3/8), DR family (FAS, TNFR1 and NGFR), Bcl-2 family (BAK, BCL2 and BCL2L1) and p53 family, but other previously seldom recognized/unrecognized apoptotic proteins, such as AMPK, DAPK3 (ZIPK), CDK7, MAPK11, β-ARK, MAPK3, RASK, and RFXK (The unrecognized standards were confirmed by the consensus results from GO annotation and PubMed search). Thus, the core apoptotic network involved in classical and novel hub proteins may provide not only a high-priority list of the key apoptotic regulators, but potential new drug targets (Figure 2B). Next, we identified several classical hub proteins such as caspases, DRs, Bcl-2 family and p53 family that have been well-characterized to play their pivotal roles in apoptosis. According to different subcellular localizations, we could divide them into secreted, nucleus, cytoplasm, multiple and unknown localizations (Figure 2B). In addition, we found that these classical hub proteins could interact with some previously unrecognized (other) hubs in the context of apoptosis (Figure 2C).

Besides the novel interactions among classical proteins, we focused on exploring the interactions between classical and other hub proteins (other hub proteins were defined by themselves with unexpectedly high levels of connectivity to apoptotic proteins) (Figure 2D). Based on 757 apoptotic hub proteins, we demonstrated that AMPK (the top one with the number of links to other hub proteins) could interact with 87 hub proteins and ZIPK (the top two with the number of links to other hub ones) could interact with 76 (see in Table S3 and Table S4). Interestingly, they could regulate the common 67 hub proteins (occupying about 72.04% of all the hub proteins they interact with) in the apoptotic kinase subnetwork (occupying about 12.29% of all hub proteins in the apoptotic kinase network), suggesting AMPK and ZIPK may be regarded as the common double targets in cancer.

### Modeling, docking and anti-proliferative activities of candidate compounds targeting AMPK/ZIPK

We firstly constructed the molecular modeling of AMPK and ZIPK based upon their crystallographic structures. Then, we screened the structure-based candidate small molecules that could target AMPK and ZIPK based on the FDA-approved and ongoing experimental drugs from Drugbank and ZINC, respectively. Subsequently, we achieved the top ten small molecule compounds from Drugbank and ZINC that could bind their target AMPK stably (Figure S2). In addition, we achieved the top ten small molecule compounds from Drugbank and ZINC that could also bind their target ZIPK stably (Figure S3). Thus, we used the top ten compounds from Drugbank and ZINC for further analyses. Then, we obtained 10 compounds by the commercial purchase or chemical synthesis named A1-A10. Then, the MTT assay was carried out in the context of HeLa and C4-I cells. Amongst all the candidate small molecules, compound A1 bear remarkable anti-proliferative activity toward these two types of cervical cancer cells in a dose-dependent manner.

### Chemical synthesis of a novel dual-target activator (BL-AD008)

**Figure 3:**
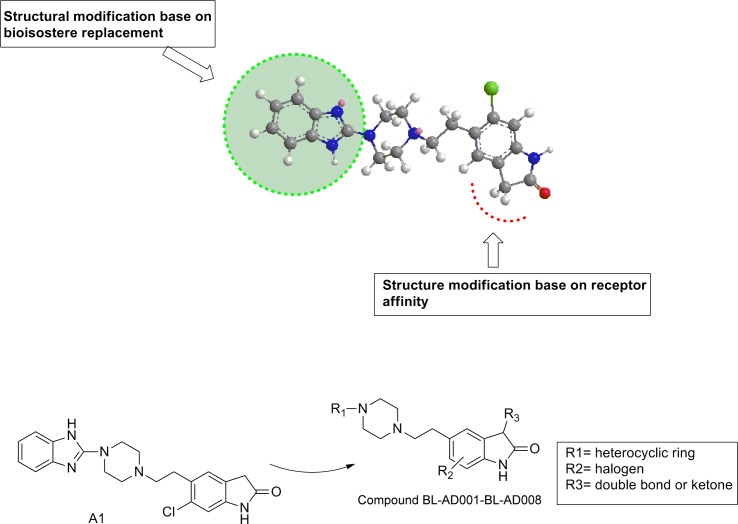
Chemical structure modification strategies of from compound A1 to BL-AD008 The structural modification was mainly focused on C-3 position of indole ring using bioisostere replacement strategy. The synthesis process of candidate compounds (AD001-BL-AD008) is described as follows. The high degree of symmetry in these molecules enabled facile confirmation by NMR technique.

Through comparing the two receptor affinity, we found that the C3-position of indole ring needed more hydrophobic interaction. Therefore, the structural modification was mainly focused on the C-3 position of indole ring using bioisostere replacement strategy (Figure 3). The synthesis process of candidate compounds (AD001-BL-AD008) was described (see in Table S5). Substituted indolones could react with chloracetyl chloride in the presence of aluminum chloride, substituted indolones could react with chloracetyl chloride to produce Friedel–Crafts reaction product 5-(2-chloroacetyl) indolinones. And, the yielding product was condensed with sodium borohydride and trifluoroacetic acid to give the reduced products 5-(2-chloroethyl) indolinones in a total yield of 43-67%. The condensation of 5-(2-chloroethyl) indolinones intermediated with heterocyclic substituted piperidine derivatives in the presence of NaI and Na_2_CO_3._ In refluxing, dioxane gave the adduct, which was finally purified by silica-gel column chromatography using hexane and ethyl acetate as an eluent to obtain the final products AD001-AD005 (yield: 45-57%) (Figure S4, Scheme 1). The syntheses of AD006-BL-AD008 were similar to the aforementioned steps with different starting materials (Figure S4, Scheme 2&3). The high degree of symmetry in these molecules enabled facile confirmation by NMR techniques. For example, in the ^1^H-NMR spectrum, the aromatic ring proton generated was observed the resonance signal at 7.08-7.97(m) which was clearly distinguishable from the resonances arising from the carbon linkers at 2.64 (m) and 2.47 (m) ppm. The purity of all compounds was above 97.0% determined by HPLC normalization method (A Waters XTerra RP18 column was eluted at flow rate of 1.0 mL/min. The mobile phase was a mixture of water and methanol containing 0.1% triethyl-amine (60:40). The eluate was monitored in the absorption at 254 nm with a UV detector). Moreover, the molecular weight of the desired target structures was confirmed by ESI-TOF high resolution mass spectrum (HRMS). Compound BL-AD008 (5-(2-(4-(benzo[d]isothiazol-3-yl)piperazin-1-yl)ethyl)-3-benzylidene-6-chloroindolin-2-one): ^1^H NMR (400 MHz, CDCl3) δ 8.89 (s, 1H), 8.54 – 8.35 (m, 1H), 8.19 – 8.03 (m, 1H), 7.94 – 7.13 (m, 10H), 3.88 (t, J = 10.2 Hz, 4H), 3.44 (t, J = 10.2 Hz, 4H), 2.80 – 2.66 (m, 2H), 2.63 – 2.47 (m, 2H). ^13^C NMR (100 MHz, CDCl3) δ 170.37, 157.23, 139.49, 135.94, 134.92, 134.39, 130.73, 130.14, 129.62, 129.45, 128.95, 128.45, 127.19, 125.88, 125.06, 122.87, 119.74, 110.66, 56.15, 52.34, 47.77, 31.32. HRMS (ESI-TOF): calcd. for C21H21N4O2S ([M+H]^+^) 501.1516, obsd. 501.1520. HPLC: 98.9%.

### Molecular docking and molecular dynamic (MD) stimulations of BL-AD008 with AMPK/ZIPK

**Figure 4:**
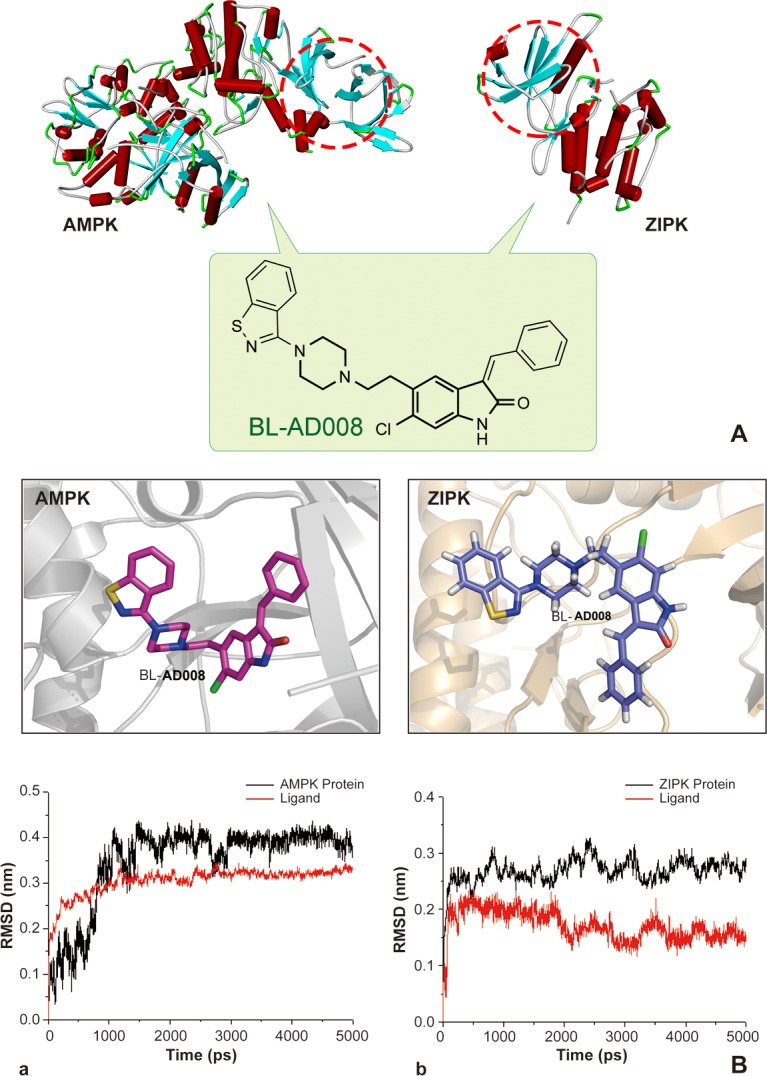
Modeling, docking and molecular dynamics (MD) simulations of BL-AD008 targeting AMPK and ZIPK

Molecular docking calculations were first performed for BL-AD008 without explicit active site water molecules. The ligand structures with the most favorable binding free energies and reasonable orientations were selected as the optimal docked conformations. 5ns MD simulations were successfully performed on AMPK–BL-AD008 and ZIPK-BL-AD008 complexes (Figure 4A). To gauge whether the MD simulations were stable and whether they converged, energetic and structural properties were monitored during the course of MD simulation. The low root-mean-square deviation (RMSD) fluctuations and the convergence of the energies, temperatures, and pressures of the systems observed indicated well-behaved systems. The RMSDs between the complexes and ligand structures obtained during the trajectories and the initial structures were shown (Figure 4B). The averaged RMSD during the last 3 ns for AMPK–BL-AD008 and ZIPK-BL-AD008 are 0.18 and 0.16 nm, respectively, suggesting the overall stable structures after approximately 3 ns simulation. In this study, we showed that compound A1 could bind to AMPK stably. The benzene ring of compound form interaction with residue LYS-31, and also form hydrophobic interaction with VAL-11, LEU-18, PHE-90 and ILE-46. For compound BL-AD008, there are two hydrogen bonds formed by residue ASN-48 and LYS-31., Cl atom of BL-AD008 could form interaction with PHE-90. BL-AD008 can form hydrophobic interaction with residue VAL-11, LEU-18, PHE-90, ILE-46 and VAL-24. Thus, the binding energy of BL-AD008 is much lower than A1 (Figure 4).

Moreover, we showed that compound A1could bind to ZIPK stably. And, there are two hydrogen bonds formed. The first one is N atom from compound A1 with carbonyl group of residue GLU-94, the other one is carbonyl group of compound A1 with N atom from main chain of residue VAL-96. A1 also form significant hydrophobic interaction with residue ILE-160, MET-146, VAL-27, VAL-96, LEU-93 and LEU-19. These residues form a hrdrophic pocket. BL-AD008, as the same as A1, two hydrogen bonds also could be found from BL-AD008 with carbonyl group of residue GLU-94 and carbonyl group of compound BL-AD008 with N atom from main chain of residue VAL-96. But comparing with A1, except form the hydrophobic interaction with the hydrophobic pocket formed by residue ILE-160, MET-146, VAL-27, VAL-96, LEU-93 and LEU-19, benzene ring of BL-AD008 formed additional more hydrophobic interaction with residue LEU-93 and LEU-68 (Figure 4). Further insights into the forces involved in substrate binding can be obtained by analyzing the MM/GBSA free-energy contributions. The binding free energies of two complex systems were calculated by MMPBSA.py program in AMBER 12 at the atomic level. A total of 300 snapshots were taken from the last 3ns of MD simulations for analysis. We present the predicted and experimental binding energies, together with their respective entropic contributions (Table S6). As displayed, the ranking of the predicted binding free energies are in good agreement with the experimental data. It should be noted that the binding free energies might not reproduce the absolute experimental values accurately, but they correlate with the experimental values well. The order of the preferentially favorable binding free energy contribution was AMPK > ZIPK, with the corresponding ΔG_*bind*_ values −40.12 and −34.18 kcal/mol, respectively. In the two studied protein-ligand systems, the van der Waals ($\Delta \;{\text{E}}_{int}^{vdw}$) contributions and the nonpolar solvation energies ($\Delta \;{\text{G}}_{\mathrm{sol}}^{\mathrm{nopol}}$) are main form for favorable binding free energies. The favorable Coulomb interactions within the systems are counteracted by the unfavorable electrostatics of de-solvation. The resulting balance of the electrostatic interaction contributions in vacuum and solvent, namely $\Delta {\text{E}}_{\mathrm{int}}^{\mathrm{ele}}+\Delta {\text{G}}_{\mathrm{sol}}^{\mathrm{ele}}$ is unfavorable in binding for all the systems.

### BL-AD008 induces apoptosis via the death receptor and mitochondrial pathways in cervical cancer cells

**Figure 5:**
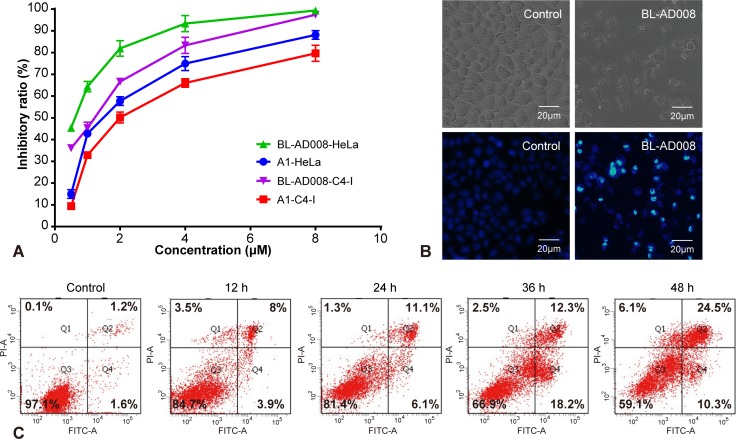
BL-AD008 induces apoptosis in HeLa cells (A) Cell viability was measured by the MTT assay in A1-treated and BL-AD008-treated HeLa cells. (B) The cellular morphology was observed without or with BL-AD008 under the inverted microscopy and fluorescent microscopy, respectively. (C) Apoptosis was determined by the analyses of Annexin staining.

We found that BL-AD008 caused a remarkable anti-proliferative effect on HeLa and C4-I cell growth in dose-dependent manner, and the treatment with 600 nM BL-AD008 for 24h resulted in almost 50% inhibition in the HeLa cells (Figure 5A). To characterize the BL-AD008-induced HeLa cell apoptosis, we observed the morphologic changes in the cells. When the cells were cultured with 600nM BL-AD008 for 24 h, the apoptotic alterations were also observed under the inverted microscopy. And, the marked apoptotic morphologic alterations were observed by Hoechst 33258 staining under fluorescence microscopy (Figure 5B). In addition, apoptosis was further evaluated by Annexin-V/PI double staining. BL-AD008 markedly induced the increase of apoptotic ratio in HeLa cells (Figure 5C).

**Figure 6:**
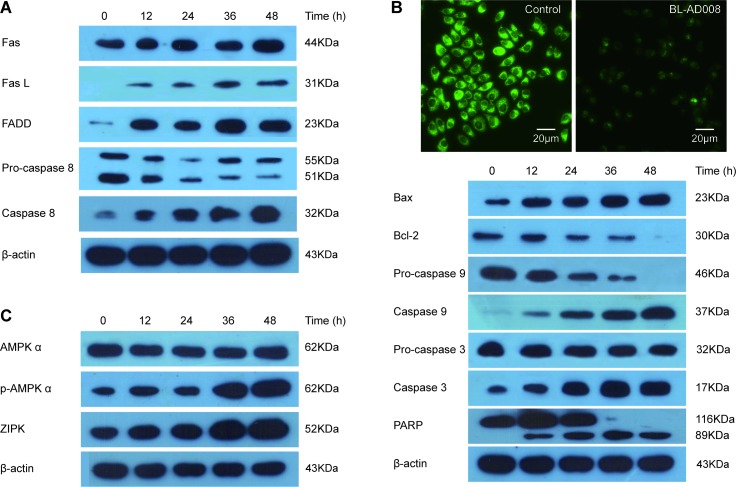
BL-AD008 induces HeLa cell apoptosis via both death-receptor and mitochondrial pathways (A) BL-AD008-induced apoptosis is via death-receptor pathway. (B) BL-AD008-induced apoptosis is via mitochondrial pathway. (C) BL-AD008-induced apoptosis is regulated by AMPK and ZIPK.

To assess whether Fas-mediated pathway was activated in BL-AD008-treated HeLa cells, the levels of Fas, FasL, Fas-Associated protein with Death Domain (FADD) and caspase-8 were determined by Western blot analysis. The levels of Fas, FasL and FADD were markedly elevated and then there was obvious increase in the cleavage of caspase-8 after BL-AD008 administration (Figure 6A). Therefore, death receptor pathway is involved in BL-AD008-induced apoptosis. Next, we found that Bax expression was increased whereas Bcl-2 expression was decreased in HeLa cells. Moreover, we detected decrease of mitochondrial membrane potential by Rhodamin 123 staining in BL-AD008-treated HeLa cells (Figure 6B). It clearly indicates that BL-AD008-induced apoptosis in HeLa cells is mediated by a mitochondrial pathway. Then, we investigated the involvements of caspase-9 and caspase-3 in BL-AD008-induced apoptosis. Caspase-9 activation was determined by measurement of the active forms of caspase-9. The active form of caspase-3 was observed during BL-AD008 treatment (Figure 6B). These results suggest that mitochondrial pathway is also involved in BL-AD008-induced apoptosis. Moreover, we showed that the ratio of p-AMPK expression was increased in BL-AD008-treared HeLa cell apoptosis, and the ratio of ZIPK expression was also increased in this context. Thus, these results suggest that BL-AD008-induced apoptosis can be mainly affected by AMPK and ZIPK in HeLa cells (Figure 6C).

### BL-AD008-induced apoptosis is mainly affected by AMPK and ZIPK

**Figure 7:**
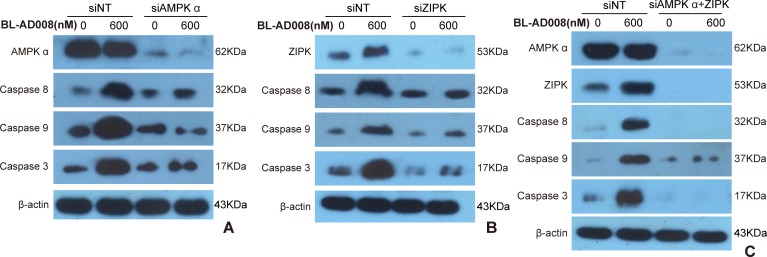
BL-AD008 induces apoptosis by targeting AMPK and ZIPK (A) BL-AD008-induced apoptosis is partly dependent on ZIPK. (B) BL-AD008-induced apoptosis is partly dependent on AMPK. (A) BL-AD008-induced apoptosis is mainly dependent on ZIPK and AMPK.

To examine whether BL-AD008 is targeted AMPK/ZIPK activator, we showed that AMPK expression was remarkably decreased in BL-AD008+AMPK siRNA-treated HeLa cells compared with BL-AD008-indcued HeLa cells. In addition, we examined the expressions of caspase-8, caspase-9 and caspase-3 between BL-AD008-treated and BL-AD008+AMPK siRNA-treated conditions. Caspase-8, caspase-9 and caspase-3 expressions were partially decreased in BL-AD008+AMPK siRNA-treated than BL-AD008-treated HeLa cells. It suggests that AMPK activation may have more effects on the activation of caspase-8, caspase-9 and caspase-3 in BL-AD008-induced apoptosis (Figure 7A).

Next, we showed that ZIPK was almost not expressed in BL-AD008+ZIPK siRNA-treated HeLa cells; whereas ZIPK was expressed in BL-AD008-indcued context. Then, we examined the different expressions of caspase-8, caspase-9 and caspase-3 between BL-AD008-treated and BL-AD008+ZIPK siRNA-treated conditions. Caspase-8 expression abruptly decreased in BL-AD008+ZIPK siRNA-treated than BL-AD008-treated HeLa cells. This result is similar with BL-AD008+AMPK siRNA-treated HeLa cells (Figure 7B). Then, we found that caspase-8, caspase-9 and caspase-3 could not be expressed in BL-AD008+ZIPK siRNA + AMPK siRNA-treated HeLa cells; whereas they could express in BL-AD008-indcued HeLa cells, suggesting that BL-AD008 may be a targeted AMPK/ZIPK activator (Figure 7C).

### BL-AD008 displays a potent anti-tumor activity *in vivo*

**Figure 8:**
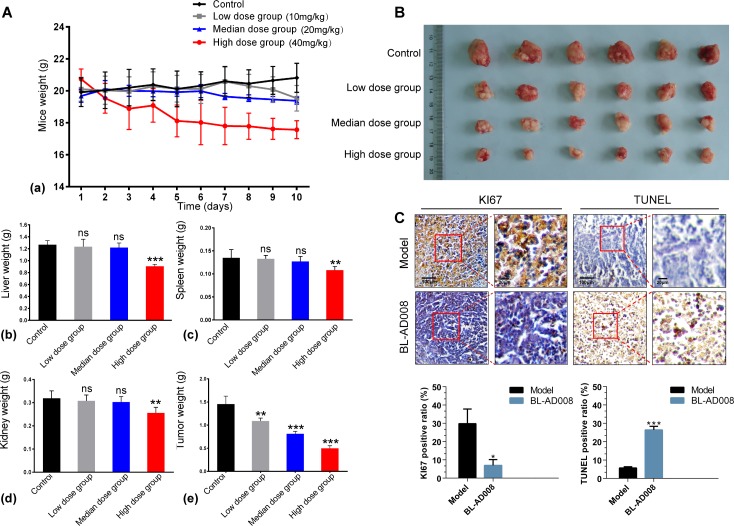
Anti-tumor effects of BL-AD008 *in vivo.* (A) Anti-tumor activities of BL-AD008 and its toxicity. The treatments began on day 1 after grouping (day 0), including vehicle, low dose of BL-AD008 10 mg/kg once a day, median dose of BL-AD008 20 mg/kg once a day and high dose of BL-AD008 40 mg/kg once a day for 10 days. Points, mean of tumors; bars, standard deviation. *, P <0.05; **, P <0.01; ***, P<0.001 compared with vehicle-treated tumors. (B) The inhibitory rate of tumor. Representative tumors from mice after vehicle and BL-AD008 treatment. (C) (a) Immunohistochemistry of proliferative marker KI67. Ki-67 expression in representative tumor section of a control mouse and a mouse of the median dose group (×100 magnification). (b) TUNEL immunohistochemistry. TUNEL immunohistochemistry in representative tumor section of a control mouse and a mouse of the median dose group (×100 magnification).

Based upon the anti-proliferative efficacy of BL-AD008 on HeLa cells *in vitro*, we proceeded to assess its efficacy on inhibiting tumor growth in an orthotopic xenograft mouse model of cervical cancer. In this experiment, we used three different doses of BL-AD008. Compared with the control group, high dose of BL-AD008 can induce the significant body weight loss in nude mice. As a result, high doses of BL-AD008 induced 15.6% loss of mice weight during the 10 days of treatment. The toxicity of low and median dose BL-AD008 were not obvious (Figure 8A). At the end of the experiment, the tumor weights decreased remarkably in median and high dose groups (P<0.001). For more toxicity study, the liver weights decrease of mice in high dose group were measured (P<0.001). And spleen and kidney weights also affected by high dose of BL-AD008 (P<0.01), no other obvious toxicity was observed in low and median dose groups. We obtained identical results by directly measuring the tumor volumes. In all three BL-AD008 groups, the tumor volumes were much smaller than the control group (Figure 8B). In according to the balance between anti-tumor efficacy and toxicity, the median dose was used as the optimum dose for treatment of tumor growth. To test whether BL-AD008-mediated inhibition of HeLa xenograft growth *in vivo* was associated with reduced cell proliferation and/or increased apoptosis, tumor tissues from control and BL-AD008-treated mice were processed for immunohistochemical analysis of Ki-67 expression and TUNEL staining. Immunoreactivity for Ki-67, a marker of proliferation, was localized to the cell nuclei. BL-AD008 treatment significantly reduced the number of Ki-67-positive HeLa cells compared to the control treatment. In addition, BL-AD008 administration resulted in a statistically significant increase in number of apoptotic bodies in the tumor as visualized by TUNEL assay, suggesting that BL-AD008-induced tumor cell proliferation inhibition through an apoptosis pathway (Figure 8C).

### BL-AD008 induces apoptosis *in vivo*

**Figure 9:**
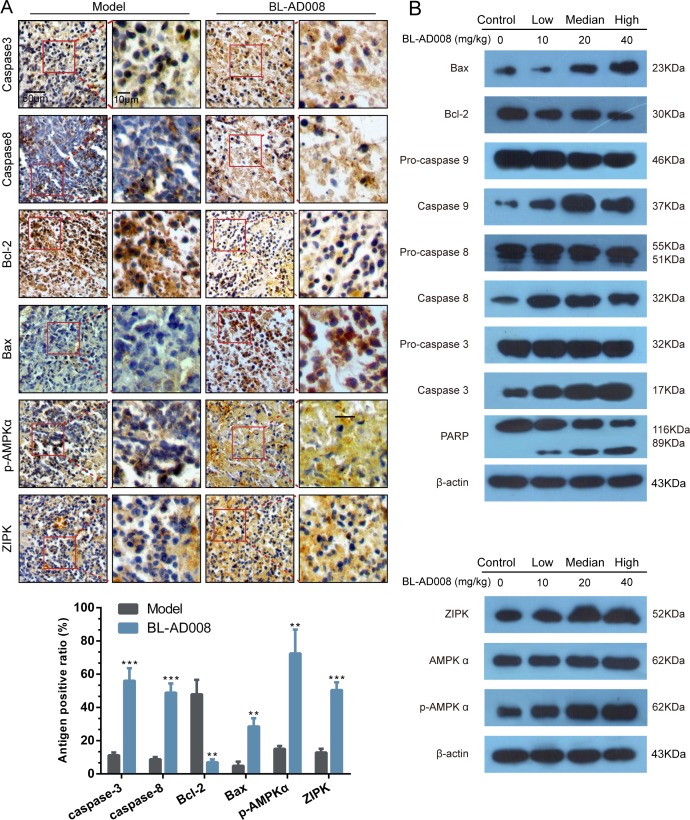
BL-AD008 induces apoptosis *in vivo* (A) Immunohistochemistry of cleaved caspase-3 and -8, Bcl-2, Bax, ZIPK, p-AMPKα. IHC staining of the mouse orthotopic tumor tissues. IHC was used to determine the expression levels of apoptosis markers, which are cleaved caspase-3 and -8, Bcl-2 and Bax. And the ZIPK, p-AMPKα levels increasing (×200 magnification). Tumor tissues excised from the median dose group treated mice; *, P <0.05; **, P <0.01; ***, P<0.001. (b) Western blot analysis of AMPK, ZIPK, ERK1, cleaved caspase-3, -8 and -9. Tumor tissues excised from the HeLa xenograft mice were lysed.

For better understanding of the mechanism of the therapeutic efficacy of BL-AD008 in our *in vivo* model, we examined the caspase-3, caspase-8, Bcl-2, Bax, p-AMPKα and ZIPK expressions in tumor samples immunoreactivity. Active form of caspase-3 and caspase-8 were observed in the tumor. And, Bcl-2 was inhibited by BL-AD008 while Bax expression was increased (Figure 9A). They were determined in tumor xenografts as parameters for the apoptosis levels. Western blotting assay further demonstrated that all their expression in BL-AD008-treated tumor samples were consistent with immunohistochemical results and caspase cascade activating. And we also tested the p-AMPKα and ZIPK for their regulation by BL-AD008. In addition, DNA repairing protein PARP-1 were sheared significantly (Figure 9B). The increase of the expression levels of p-AMPKα and ZIPK confirmed the efficacy of BL-AD008 in tumor tissues. Moreover, immunohistochemical and western blotting results suggesting the apoptosis induced by BL-AD008 in tumor tissues.

## DISCUSSION

Previous studies reported that the cancer-perturbed PPI network was well-characterized by a nonlinear stochastic model, maximum likelihood parameter estimation, and akaike information criteria for the discovery of novel apoptotic targets, based upon the gene co-expression profiling [17]. Other reports demonstrated that the central apoptotic pathways and their connections were built by a large-scale literature-based Boolean model, which may assume that the apoptotic pathways are either present or absent (on/off) [18]. These mathematics models can uncover some core pathways s in the apoptotic process; however, the PPI network is actually complex, non-linear and not simply composed of on/off connections or depends on solely biological evidence. The PPI network is typically complicated for its nature, with multiple connections amongst numerous signaling pathways; thus, it is necessary to represent this network by using the Naïve Bayesian model that can integrate disparate data types into an advantageous platform [19]. We developed Naïve Bayesian model, which was well-suited to integrate these high-throughput data such as SSBP, gene co-expression profiles, DDI and cross-species interolog mapping for predicting protein functional connections; thereby, constructing the apoptotic PPI network. Moreover, we used a multiple analysis method which could integrate four golden standards such as degree, GO annotation, network module and microarray analysis for identifying our apoptotic hub proteins. Compared to previous studies for the discovery of novel apoptotic targets based on sole evidence [17, 18], we used our multiple analysis which may include more biological characteristics and thus being more accurate for apoptotic hub protein/target identification.

Previous studies have demonstrated that a mechanistic mathematical model can describe the temporal evolution of caspase activation, indicating the key elements of receptor-modulated caspase activation [20, 21]. Another study has reported that prediction of caspase cleavage sites can be carried out by Bayesian bio-basis function neural networks [22]. In this study, we computationally predicted that caspase-3 and -8 were involved in the core apoptotic network, and subsequently confirmed that caspase-8, -9 and -3 played their roles in BL-AD008-treated HeLa cells. Apoptosis is triggered by the extrinsic (death receptor) or intrinsic (mitochondrial) pathway. Death receptor pathway can be initiated by stimulations of members of DR family such as CD95/Fas, TRAIL, and TNF [20]. Previous studies have shown that a model can characterize apoptosis initiation at the CD95 DISC, considering activation of procaspase-8 at the DISC and inhibition of procaspase-8 activation by c-FLIP proteins. CD95 signaling defines a threshold activation behavior, and the decisions to undergo apoptosis depend on the ratio between procaspase-8 and c-FLIP proteins [23]. Another model of the cross-talks between CD95-mediated apoptotic and non-apoptotic signaling have been reported that life/death decision is taken at the DISC and defined by DED protein concentrations and c-FLIP cleavage product generation [24]. Other studies have demonstrated that death receptors play the key roles in deciding the apoptotic network and adding to signal processing capabilities attributed to receptor clustering [25, 26]. Besides, caspases are linked to Bcl-2 family which is the key regulator of apoptosis and often over-expresses in cancer [27, 28]. Therefore, we showed the close relationships among caspases, DRs and Bcl-2 family, indicating that the apoptotic PPI network is high-dependable; thus, providing a basic framework of apoptosis. Recently, a model of mitochondrial pathways has been reported that apoptosome-dependent caspase activation depends on the concentration of XIAP, indicating the key roles of procaspase-9 and caspase-3, as well as of a positive feedback loop between caspase-3 and caspase-9 [29]. In this study, we computationally predicted some death receptors and Bcl-2 family members that were both involved in the apoptotic subnetwork. Subsequently, we experimentally validated that BL-AD008-induced apoptosis was dependent on both death receptor and mitochondrial pathways.

Interestingly, we identified not only the aforementioned classical hub proteins such as caspases, DR family and Bcl-2 family that can be implicated in core apoptotic pathways, but some ‘novel’ hub proteins/targets, such as AMPK and ZIPK in core apoptotic pathways. Accordingly, we screened the above-mentioned hub proteins that may provide a ranked list of high-priority, new kinase targets. Previous study has reported that targeting PI3KCI/Akt/mTOR signaling with inhibitors such as ATP-competitive compounds can improve cancer therapeutic effect because the catalytic sites of PI3KCI and mTOR share a high degree of sequence homology [30]. In addition, other report has demonstrated that the dual PI3KCI/mTOR inhibitor PI-103 and the Mdm2 inhibitor Nutlin-3 is a combination strategy aimed at inhibiting PI3KCI/Akt/mTOR signaling and activating p53 signaling in AML [31]. Moreover, a recent report has shown that ZD6474 can evaluate the feasibility and efficacy of combined VEGFR2 and EGFR *in* breast cancer cells, which may be an alternative approach to the ongoing conventional cancer radiotherapy [32]. Distinctive from these dual-target strategies, we decided to target both AMPK and ZIPK by systems biology network prediction, which provided the clue that the two kinase could interact with many apoptotic kinases and thus play their crucial roles in the apoptotic kinase subnetwork of cancer. It is well-known that AMP-activated protein kinase (AMPK) is a serine/threonine protein kinase, serving as an energy sensor, and its activation strongly suppresses cancer cell proliferation. Zipper interacting protein kinase (ZIPK), also known as death associated protein kinase 3, is a serine/threonine kinase that mediates apoptosis in cancer cells. Of note, resisting cell death and deregulating cellular energetics are the two hallmarks of cancer. Besides, they have high similarity (54.3%) in their kinase domains. But, there are differences in some amino acid sites which make their difference in the tertiary structures. Thus, simultaneously targeting the two kinases may be a promising avenue for killing two birds with one stone.

Based upon the two target identification and their similarity in kinase domains, we synthesized a series of candidate compounds and found a novel small molecule activator (BL-AD008), and identified this activator could induce cervical cancer cell apoptosis by the death-receptor and mitochondrial pathways. Moreover, in our study, we found that BL-AD008 bear the good anti-tumor activities without remarkable toxicities, and also induced apoptosis by targeting AMPK/ZIPK *in vivo*. In our study, we found that ZIPK may play more important role than AMPK by siRNA experiments, suggesting that apoptosis may be main target for BL-AD008 in cervical cancer therapy.

**Figure 10:**
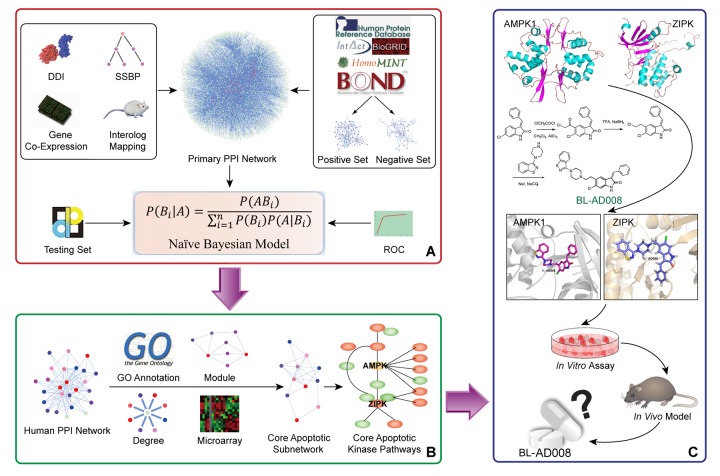
A schematic model of network prediction and experimental validation of a novel AMPK/ZIPK activator BL-AD008 in cervical cancer (A) We systematically constructed the global protein-protein interaction (PPI) network and integrated four different biological evidence to predict apoptosis-related protein connections by the Naïve Bayesian model; (B) Utilizing the Naïve Bayesian model and four golden standards, we identified some key apoptotic kinase targets, such as AMPK and ZIPK in cancer; (C) We screened many candidate compounds, synthesized some compounds and eventually designed a novel dual-target activator (BL-AD008) that induced the death-receptor and mitochondrial apoptosis, which was affected by AMPK and ZIPK in cervical cancer *in vitro* and *in vivo*.

In conclusion, we demonstrate the ability of our Naïve Bayesian model-based network for identifying the key double targets AMPK and ZIPK, and provide the dual-target activator (BL-AD008) as a potential new apoptosis-modulating drug for cervical cancer therapy (Figure 10). Therefore, these findings would lead to a comprehensive mechanistic insights into identification of more ideal dual targets as well as discovery of more new kinase activators. Moreover, it would also provide a basis for developing more new systems biology network-based approaches and more promising strategies for future cancer therapeutics.

## MATERIALS AND METHODS

### Retrieving functional genomics data

Diverse sets of biological evidence were collected from several online databases to build the global PPI network. To predict pair-wise protein–protein relationship, all the data were preprocessed into pair-wise scores, reflecting the similarity between protein pairs. And, five online databases included protein interaction data from Human Protein Reference Database (HPRD) [33], Biomolecular Object Network Databank (BOND) [34], IntAct [35], HomoMINT [36] and BioGRID [37]. Thus, Gold Standard Positive (GSP) interaction set was constructed by these online databases. Gold Standard Negative (GSN) interaction set was defined through protein pairs in which one protein was from the plasma membrane cellular componentand the other was from the nuclear cellular component, as assigned by Gene Ontology (GO) Consortium. 23,169,177 unique pairs, in total, were identified except for 5,275 overlapping pairs with GSP. Additionally, the data in Standard Test Set (STS) were retrieved from Database of Interacting Proteins (DIP) [38] and matched randomly by these proteins, and apoptotic proteins were from GO annotation. Raw data were constructed by randomly matching amongst all the human proteins in UniProt database.

### Multiple sources of biological data

**Gene co-expression profiles:** Proteins that can interact with each other often possess similar gene expression patterns; thereby, genes that can co-express should be more likely to interact than genes that cannot co-express. To identify genes that are co-expressed, we used microarray data of HeLa cells and primary human lung fibroblasts treated with 2.5mM DTT to measure the pair-wise co-expression level of related genes in apoptosis [39]. The co-expression level is calculated as Pearson Correlation Coefficient ρ

$$\rho_{X,Y}\;=\;\frac{\sum_{i=1}^{n}\left(X_{i}\;-\;\bar{X}\right)\left(Y_{i}\;-\;\bar{Y}\right)}{\left(n\;-\;1\right)\sigma_{X}\sigma_{Y}}$$

Where *X* and *Y* are expression level data vectors of length n for two genes, $\bar{X}$ and $\bar{Y}$ are means, and σ_X_ and σ_Y_ are the standard deviations.

**Domain-domain interaction (DDI):** Because protein interactions involve physical associations between protein domains, it is proposed that novel protein interactions may be predicted by identifying the pairs of domains enriched amongst known interacting proteins. To test this logic into the context of our GSP and GSN sets, domain-domain interaction relationships were downloaded from Pfam [40].

**Cross-species interolog mapping:** The human orthologs of model organism proteins often retain similar function; therefore, pair of human orthologs that interact in a model organism are likely to interact in human. Model organisms [*Caenorhabditis elegans* (4,649), *Drosophila melanogaster* (5,527), *Saccharomyces cerevisiae* (2,154), *Rattus norvegicus* (15,306), *Mus musculus* (16,376), and *Escherichia coli* (541)] were mapped into human protein pairs, by gene orthologs defined in the Inparanoid database by clustering into orthologous groups.

**Smallest shared biological process (SSBP):** Interacting proteins often function in the same biological process, and proteins functioning in small, specific processes should be more likely to interact than proteins functioning in large, general processes. The procedure was used to quantify functional similarity between two proteins: 1) to identify all biological process terms shared by two proteins; 2) to count how many other proteins were assigned to each of the shared terms; 3) to identify the shared biological process term with the smallest count. In general, the smaller and the more specific is the biological process term, which indicates the greater functional similarity between two proteins. Protein pairs were binned by this measure of functional similarity and then the degree of similarity was tested for its ability to predict PPIs.

### Integration and evaluation of biological data into the Naïve Bayesian model

We develop a Naive Bayesian model to integrate diverse data and make the final interaction predictions in an integrated way [41]. Following the Bayesian theorem, we compute the posterior odds given n evidence as follows:

$$O_{posterior}\;=\;\frac{P(positive\;|\;E_{1},...,E_{n})}{P(negative\;|\;E_{1},...,E_{n})}$$

Where *positive* means that two proteins are functional related while *negative* means not. We define

$$LR_{\left(E_{1},...,E_{n}\right)}\;=\;\frac{P(E_{1},...,E_{n}\;|\;positive)}{P(E_{1},...,E_{n}\;|\;negative)}$$

then Oposterior = Oprior*LR. As Naive Bayesian model supposes that each of the evidence is conditional independent, we can simplify LR as $LR_{\left(E_{1},...,E_{n}\right)}\;=\;\prod_{i=1}^{n}LR_{\left(E_{i}\right)}$ As the prior odds is a constant, the composite LR corresponding to a type of specific biological evidence can be used to measure the predictive power or confidence degree for predicting functional links. A cutoff of likelihood ratio (LR cut) is represented as an indicator whether a protein pair bears the functional relation. Then, we filter the initial networks through Naïve Bayesian model by selecting the pairs with composite LR above the cutoff. A receiver operating characteristic (ROC) curve can elucidate the relationship between the sensitivity and specificity of a binary classifier system for different cut points [42]. The ROC curve can be represented equivalently by plotting the fraction of true positive rate (TPR) *versus* the fraction of false-positive rate (FPR). Sensitivity and specificity can measure the ability of a classifier to identify true positives and false positives in a test, and calculated as sensitivity= TP/positives, and specificity = 1 – (FP/negatives), where TP and FP are the number of true positives and false positives identified by a classifier, respectively; whereas positives and negatives are the total number of positives and negatives in a test. The area under the ROC curve is an indicator of the efficacy of the assessment system. Thus, the performances of different classifiers appear to be comparable by measuring the ROC curves, suggesting that the larger the ROC curve is; the better the performance is.

### Hub protein identification in apoptosis

We identified hub proteins implicated in core apoptotic pathways according to the following four golden standards: 1) the degree of each protein: we selected the number of degrees which is bigger than or equal to 300. 2) The link number of apoptotic protein: we choose the number of links to other known apoptotic proteins that are bigger than or equal to 300 (the standard of classical hub proteins) or 200 (the standard of novel hub proteins) respectively. 3) Network topology: we suggest that hub proteins often enrich in the “dense area” rather than “sparse area” in cancer. 4) Significance analysis of microarrays (SAM) analysis is performed on data from expression microarray in apoptotic stress to identify genes with greatly divergent expressions between normal and cancer cells; thus, we indicated that the proteins, identified as divergent expression proteins that were extracted as functional hub protein.

### Modeling, docking and molecular dynamics (MD) simulations

The initial three dimensional geometric coordinates of the X-ray crystal structure of AAPK1 (AMPK, PDB code: 2V8Q) and DAPK3 (ZIPK, PDB code: 3BHY) were downloaded from the Protein Databank (PDB), respectively. And, we constructed the screening library for them containing all the small molecule compounds from the latest version of Drugbank (http://www.drugbank.ca/) and ZINC (http://zinc.docking.org/), respectively. The activators were constructed using the Accelrys Discovery Studio (version 3.5; Accelrys, SanDiego, CA, USA) molecular modeling software and were energy minimized with the CHARMm force field. Docking and MD simulation were performed according to our previous reports [43, 44]. The CDOCKER protocol was employed as docking approach to conduct semi-flexible docking. 5 ns MD simulations were carried out for AMPK-BL-AD008 and ZIPK–BL-AD008 complexes. The protein–ligand binding free energy was calculated based on 300 snapshots taken from 2 to 5 ns MD simulation trajectories of the complex.

### Chemical synthesis of candidate compounds

All reactions requiring anhydrous conditions were performed under an Ar or N_2_ atmosphere. Chemicals and solvents were either A.R. grade or purified by standard techniques. Thin layer chromatography (TLC): silica gel plates GF_254_; compounds were visualized by irradiation with UV light and/or by treatment with a solution of phosphomolybdic acid (20% wt. in ethanol) followed by heating. Column chromatography was performed by using silica gel with eluent given in parentheses. ^1^H NMR and ^13^C-NMR analysis was determined on a Bruker Avance III 400MHz spectrometer and performed using CDCl_3_ or DMSO-*d*_6_ as a solvent at room temperature. The chemical shifts are expressed in relative to TMS (=0 ppm) and the coupling constants J in Hz. The purity of compound screened in biological assays was determined to be ≥97% by HPLC (Agilent 1100 HPLC system) analysis with a photodiode array detector, An atlantis C18 (150 mm × 4.6 mm, i.d. 5μm) (Waters, Milford, Mass, USA) was used with a gradient elution of methanol and HPLC-grade water as mobile phase at a flow rate of 1 mL/min. HRMS data were obtained using Bruker micro-TOF-Q instrument or TOF-MS instrument.

### Cell culture

The HeLa and C4-I cells were purchased from American Type Culture Collection (ATCC, Manassas, VA, USA). They were routinely cultured in RPMI-1640 or Waymouth's MB 752/1 medium containing 10% fetal bovine serum, 100 U/ml streptomycin, 100 U/ml penicillin, and 2 mM L-glutamine in a humidified cell incubator with an atmosphere of 5% CO_2_ at 37 °C.

### Cell viability assay

The HeLa and C4-I cells were dispensed in 96-well flat bottom microtiter plates at a density of 5×10^4^ cells/ml. After 24 h incubation, they were treated with different concentrations of A1 and BL-AD008 for the indicated time periods, respectively. Cell viability was measured by the 3-(4, 5-dimetrylthiazol-2-yl)-2, 5-diphenyltetrazolium bromide (MTT) assay.

### Apoptosis assay

The HeLa cells were seeded into 6-well culture plates with or without BL-AD008 and cultured for 24 h, then incubated with 500 μL hoechst 33258 or rhodamin 123 staining solution in the dark at 37 °C for 30 min and observed under fluorescence microscope. Apoptotic ratio was measured by Annexin-V-FLUOS Staining Kit (Roche, Germany) according to the manufacturer's protocol followed by FACScan flow cytometry analysis (Becton Dickinson, Franklin Lakes, NJ).

### Western blot analysis

The HeLa cells were treated with 600nM BL-AD008 for 0, 12, 24, 36 and 48h respectively. Both adherent and floating cells were collected, and then western blot analysis was carried out by the method as follow. The cell pellets were resuspended with lysis buffer consisting of Hepes 50 mmol/L pH 7.4, Triton-X-100 1%, sodium orthovanada 2 mmol/L, sodium fluoride 100 mmol/L, edetic acid 1 mmol/L, PMSF 1 mmol/L, aprotinin 10 mg/L and leupeptin 10 mg/L and lysed at 4°C for 1 h. After 14,000×g centrifugation for 15 min, the protein content of supernatant was determined by the Bio-Rad DC protein assay (Bio-Rad Laboratories, Hercules, CA, USA). Equal amounts of the total protein were separated by 10-15 % SDS-PAGE and transferred to PVDF membranes, the membranes were soaked in blocking buffer (5 % skimmed milk). Proteins were detected using primary antibodies, followed by HRP-conjugated secondary antibody and visualized by using ECL as the HRP substrate.

### SiRNA transfection

Small interfering RNAs (siRNAs) against human AMPK, ZIPK and control siRNA were purchased from Invitrogen (Carlsbad, CA). The HeLa cells were transfected with siRNAs at 100 nM final concentration using Lipofectamine 2000 (Invitrogen) according to the manufacturer's instructions. The transfected cells were used for subsequent experiments 24 h later.

### Mouse experiments and tumor xenograft model

The Institutional Animal Care and Treatment Committee of Sichuan University approved all studies herein. 24 healthy female nude mice (BALB/c, 6–8 weeks of age, non-fertile and 18–20 g each) were injected subcutaneously with HeLa cells (1×10^7^cells/mouse). When the tumors reached 100 mm^3^ in volume (calculated as V = L×W^2^/2). The mice were divided into four groups. Three groups were treated with different dose groups of BL-AD008 once a day by intraperitoneal injection for 10 days (low dose group, 10 mg/kg; median dose group, 20 mg/kg; high dose group, 40mg/kg), whereas the control group was treated with vehicle control (5% CMC-Na). Body weight was determined every day until the end of the study. At the end of the treatment, all mice were sacrificed. Tumor tissue, spleen, liver and kidney were harvested, weighed, photographed, prepared for immunohistochemistry or lysed for western blotting.

### Immunohistochemical analysis and TUNEL assay

Tumor tissues obtained from *in vivo* studies were rinsed in PBS and fixed in 4% paraformaldehyde. Samples were dehydrated in gradient ethanol, paraffin embedded, and sectioned (4 μm). Deparaffinized sections were stained with primary antibodies. The samples were incubated overnight with biotinylated secondary antibodies. Detection was done with avidin-biotin-HRP complex (Thermo scientific, Fremont, CA) and DAB as chromogen. Nuclei were counterstained with hematoxylin. The positive cells were counted in six fields per tumor sample. Results are expressed as the average ± S.D. of tumors per group. For TUNEL assay, sections were permeabilized with 0.1% Trition X-100 plus 0.1% sodium citrate and then incubated with 50 ml TUNEL reaction mixture (Roche) at 37 °C for 60 min. After rinsing with PBS three times, 50 ml converter-POD was added and the tissue cells were incubated in a humidified chamber for 30 min at 37 °C. DAB substrate was then added, followed by counterstaining with hematoxylin. The assay included negative controls (without terminal transferase). Apoptosis was quantified by counting the number of TUNEL-positive cells in at least six non-overlapping high-power fields on each section and evaluated.

### Statistical analysis

All the presented data and results were confirmed in at least three independent experiments. The data are expressed as means ± S.D. Statistical comparisons were made by Student's t-test. P<0.05 was considered statistically significant.

## SUPPLEMENTARY MATERIAL, FIGURES AND TABLES








