# Supplementary material for: Systems biology network-based discovery of a small molecule activator BL-AD008 targeting AMPK/ZIPK and inducing apoptosis in cervical cancer
Source: Oncotarget. 2015 Mar 10;6(10):8071–88. doi: 10.18632/oncotarget.3513 (PMC4480736; doi:10.18632/oncotarget.3513)
Supplement: Supplementary file 1 [file oncotarget-06-8071-s001.pdf]

# Systems biology network-based discovery of a small molecule activator BL-AD008 targeting AMPK/ZIPK and inducing apoptosis in cervical cancer

## Supplementary Material

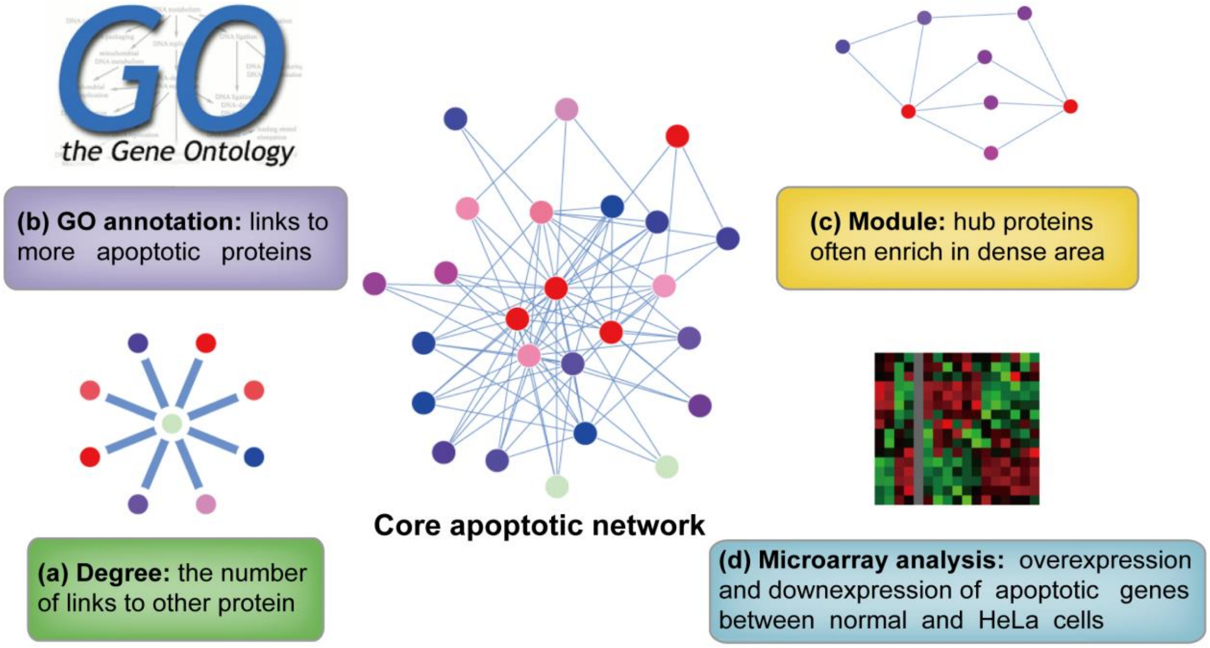


**Figure S1: Multiple analyses of the apoptotic hub proteins/targets in cancer.**

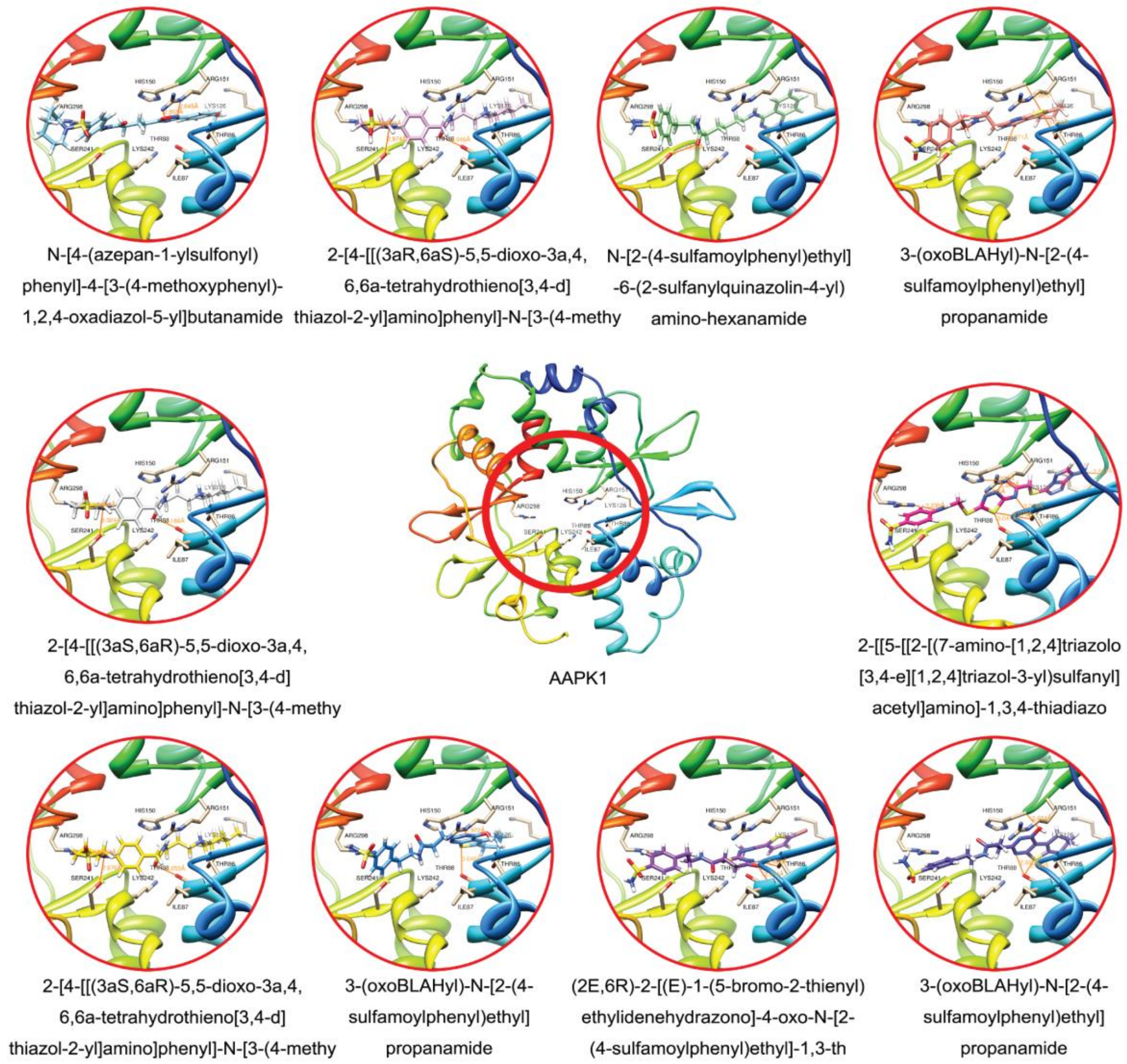


**Figure S2: The consensus results of the top ten candidate compounds targeting AMPK from Drugbank and ZINC.**

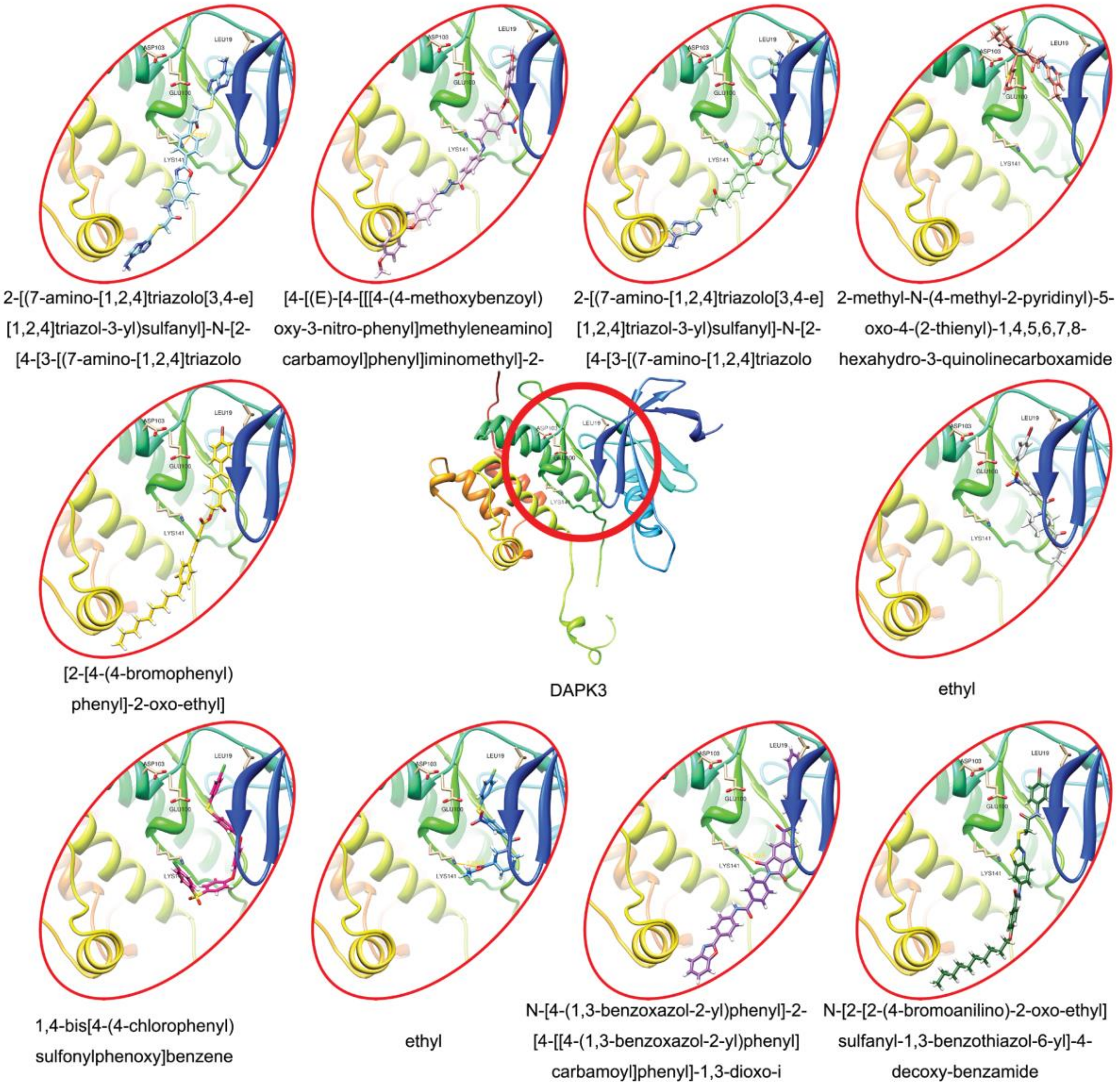


**Figure S3: The consensus results of the top ten candidate compounds targeting ZIPK from Drugbank and ZINC.**

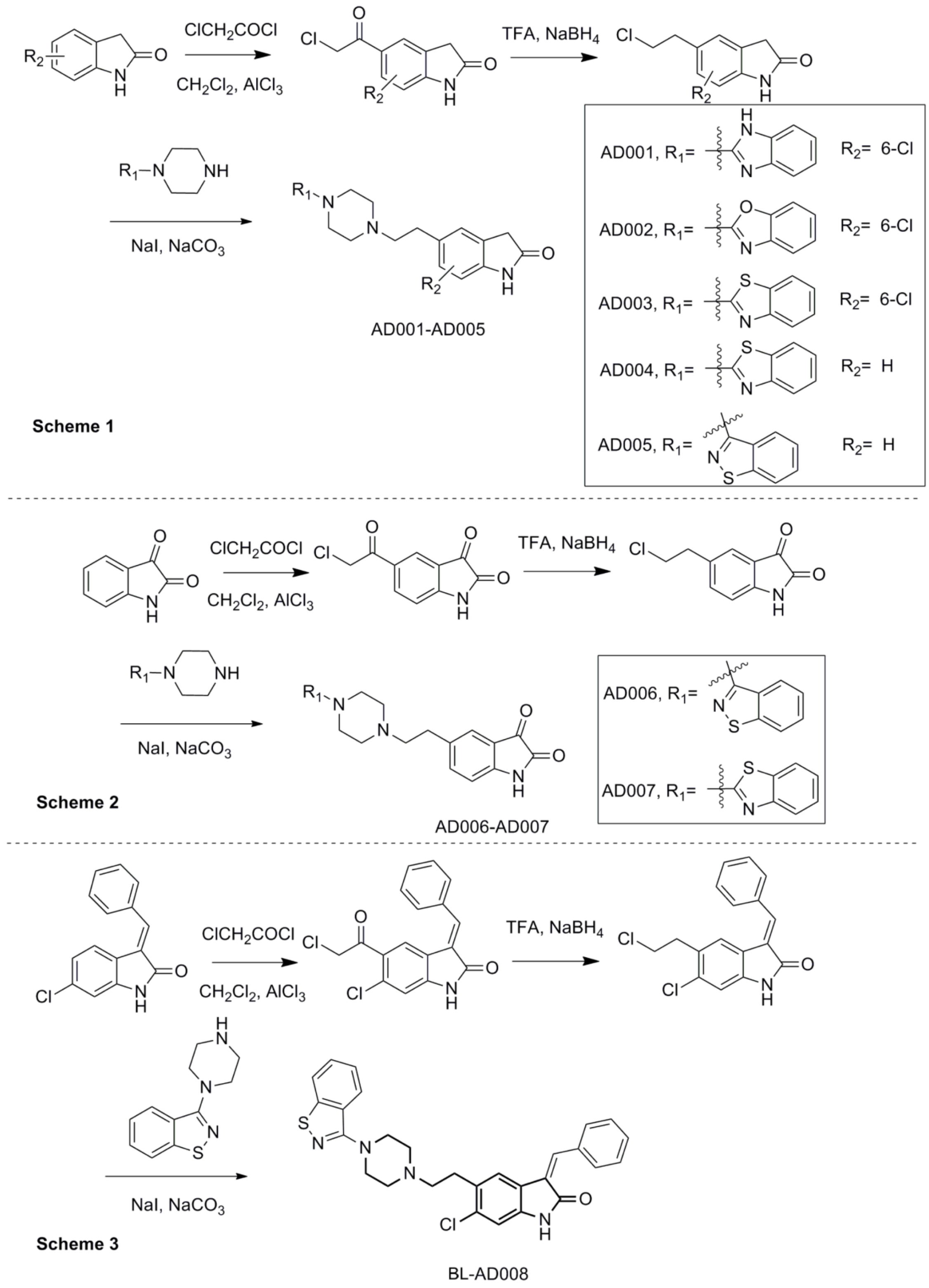


**Figure S4: The synthesis of BL-AD008 as a novel dual-target activator.**

**Table S1: Integration of data sources for predicting human protein-protein interactions.**

| Prediction type | Dataset name | Time stamping | Element number |
|---|---|---|---|
| Co-expression | Pairwise co-expression set | 2014/10/14 | 8, 030, 028 |
| Enriched domain pair | Domain-domain interaction set | 2014/11/17 | 148, 938 |
| Ortholog interactions | Interolog mapping PPI set | 2014/5/10 | 7, 090 |
| Shared biological process | Shared biological process enrichment set | 2014/10/04 | 31, 318 |
| **Gold standard** | **Data set** | **Time stamping** | **Element number** |
| Positive(GSP) | Good standard positive set | 2014/10/05 | 85, 083 |
| | Testing set | 2014/5/10 | 25, 620 |
| Negative(GSN) | Good standard negative set | 2014/5/14 | 23, 169, 177 |
| Raw pair | Raw protein-protein interaction set | 2014/5/14 | 204, 919, 890 |

**Table S2: Protein-protein interactors from core apoptotic subnetwork.**

**Table S3: The interactors with AMPK in apoptosis.**

**Table S4: The interactors with ZIPK in apoptosis.**

**Table S5: Modified candidate small molecule compounds from A1 to A10.**

| Compound | IUPAC Name | Chemical Structures |
|---|---|---|
| A-1 | 5-(2-(4-(benzo[d]isothiazol-3-yl)piperazin-1-yl)ethyl)-6-chloroindolin-2-one | 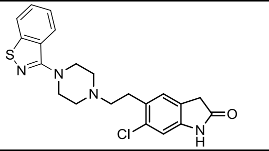 |
| AD-001 | 5-(2-(4-(1H-benzo[d]imidazol-2-yl)piperazin-1-yl)ethyl)-6-chloroindolin-2-one | 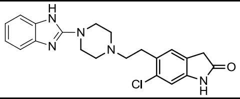 |
| AD-002 | 5-(2-(4-(benzo[d]oxazol-2-yl)piperazin-1-yl)ethyl)-6-chloroindolin-2-one | 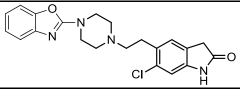 |
| AD-003 | 5-(2-(4-(benzo[d]thiazol-2-yl)piperazin-1-yl)ethyl)-6-chloroindolin-2-one | 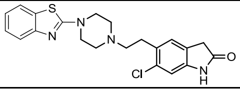 |
| AD-004 | 5-(2-(4-(benzo[d]thiazol-2-yl)piperazin-1-yl)ethyl)indolin-2-one | 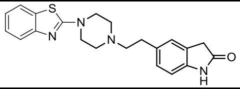 |
| AD-005 | 5-(2-(4-(benzo[d]isothiazol-3-yl)piperazin-1-yl)ethyl)indolin-2-one | 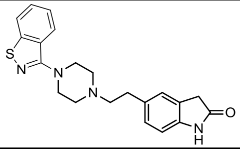 |
| AD-006 | 5-(2-(4-(benzo[d]isothiazol-3-yl)piperazin-1-yl)ethyl)indoline-2,3-dione | 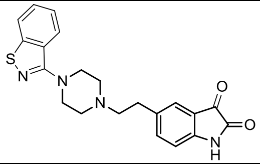 |
| AD-007 | 5-(2-(4-(benzo[d]thiazol-2-yl)piperazin-1-yl)ethyl)indoline-2,3-dione | 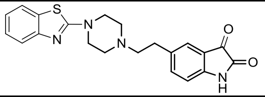 |
| **BL-AD-008** | 5-(2-(4-(benzo[d]isothiazol-3-yl)piperazin-1-yl)ethyl)-3-benzylidene-6-chloroindolin-2-one | 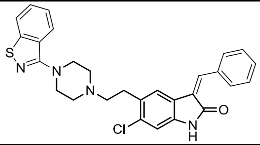 |

**Table S6: Binding free energies and individual energy terms of BL-AD008 in complex with both AMPK and ZIPK (kcal/mol).**

| Contribution | AD008-AMPK | AD008-ZIPK |
|---|---|---|
| $\Delta E_{\mathrm{int}}^{ele}$ | -7.11(0.23) | -0.91(0.04) |
| $\Delta E_{\mathrm{int}}^{vdw}$ | -38.04 (0.39) | -31.52(0.40) |
| $\Delta G_{sol}^{nopol}$ | -4.2(0.04) | -3.66(0.05) |
| $\Delta G_{sol}^{ele}$ | 21.73(0.27) | 12.82(0.56) |
| $\Delta G_{sol}$ [a] | 17.53(0.26) | 9.16(0.52) |
| $\Delta G_{ele}$ [b] | 14.62(0.38) | 11.91(0.59) |
| -TΔS | -12.7 | -10.9 |
| $\Delta G_{bind}$ | -40.12(0.41) | -34.18(0.34) |

[a] The polar/nonpolar ($\Delta G_{sol}^{ele} + \Delta G_{sol}^{nopol}$) contributions.

[b] The electrostatic ($\Delta E_{\mathrm{int}}^{ele} + \Delta G_{sol}^{ele}$) contributions. All energies are averaged over 300 snapshots and are given in kcal/mol. Calculation of $\Delta G_{bind}$ does not explicitly consider entropy contributions. The values in parentheses represent the standard error of the mean.
